# An Exploration of the Effects of an Early Postpartum Intravenous Infusion with Carnosic Acid on Physiological Responses of Transition Dairy Cows

**DOI:** 10.3390/antiox10091478

**Published:** 2021-09-16

**Authors:** Tainara Cristina Michelotti, Erminio Trevisi, Johan S. Osorio

**Affiliations:** 1 Dairy and Food Science Department, South Dakota State University, Brookings, SD 57007, USA; Tainara.Michelotti@sdstate.edu; 2 Department of Animal Sciences, Food and Nutrition (DIANA), Facoltà di Scienze Agrarie, Alimentari e Ambientali, Università Cattolica del Sacro Cuore, 29122 Piacenza, Italy; erminio.trevisi@unicatt.it

**Keywords:** peripartum, oxidative stress, carnosic acid

## Abstract

The objective of the present study was to evaluate the effects of an antioxidant and anti-inflammatory compound found in rosemary plants (*Salvia rosmarinus*) named carnosic acid during the transition period of dairy cows. From day 1 to 3 after calving, 16 multiparous Holstein cows received a daily intravenous infusion of either 500 mL of saline (NaCl 0.9%; Saline; *n* = 8) or carnosic acid at a rate of 0.3 mg/kg of BW supplied in 500 mL of saline (CA; *n* = 8). Blood samples were taken at –7, 2, 5, 7, 14, and 21 d relative to parturition, then analyzed for metabolites related to energy metabolism, muscle mass catabolism, liver function, inflammation, and oxidative stress. CA infusion tended to improve milk performance; however, DMI was unaffected by treatment. At 2 d relative to parturition, CA cows had lower blood concentrations of haptoglobin, paraoxonase, FRAP, and NO_2_^–^ than saline cows. After treatment infusions, haptoglobin remained lower in CA cows than saline at 5 d relative to parturition. Our results demonstrate that carnosic acid promoted positive responses on inflammation and oxidative stress biomarkers and may promote beneficial effects on lactation performance in peripartal dairy cows.

## 1. Introduction

The transition from pregnancy to lactation is one of the most susceptible periods of a dairy cow’s life. Approximately 75% of health disorders in adult cows, such as retained placenta, mastitis, and metritis, typically occur within the first month after calving [1]. The high susceptibility to these diseases is mainly correlated with an impaired immune response, resulting in drastic metabolic and endocrine changes during the transition period. From a metabolic standpoint, dairy cows commonly experience an increase in energy requirements during the transition period, primarily for fetal growth prepartum, followed by milk synthesis postpartum [2,3]. Then, cows show a clear inflammatory response [4,5] after calving, although changes in immune response often proceeded parturition [6,7,8]. Additionally, during the prepartum, cows commonly experience a decrease in dry matter intake (DMI) [9]. Although immediately after parturition, there is a progressive increase in feed intake, this is generally insufficient in parallel with the nutrient requirements in early lactation. This results in a negative energy balance (NEB) condition, and consequently, non-esterified fatty acids (NEFA) are mobilized from the adipose tissue [10,11].

During NEB, triacylglycerides (TAG) are hydrolyzed into glycerol and NEFA and released into the bloodstream [12]. NEFA can be used as an energy source by different tissues, such as the mammary gland for milk fat synthesis, or taken up by the liver [13]. In the liver, NEFA are either completely oxidized to generate energy, partially oxidized to produce ketone bodies, or re-esterified into TAG. Then, TAG are either exported as very low-density lipoproteins (VLDL) or stored in the hepatocytes [13,14]. At periods of intense lipid mobilization, as the transition period, NEFA entering the liver exceeds its capacity of β-oxidation and VLDL secretion, which follows to a greater production of ketone bodies, such as b-hydroxybutyrate (BHB), and the accumulation of TAG in the liver [14,15].

The increased plasma NEFA and BHB, aligned with augmented metabolic demand characteristic of the transition period, is commonly accompanied by a surge in reactive oxygen species (ROS) leading to oxidative stress [11,16]. Parallel to this, the pro-inflammatory cytokines released by the immune system induce ROS production by both phagocytic and non-phagocytic cells [11], which can further alter redox balance and lead to an oxidative stress condition during early postpartum. Oxidative stress can be defined as the damage occurring to cellular macromolecules as a consequence of serious and prolonged imbalance between oxidants and antioxidants [17,18]. ROS are particularly reactive towards DNA, proteins, and lipids, resulting in cellular alterations, including lipid peroxidation, cell membrane damage, DNA mutation, impaired protein activity, and cell death [16,18,19]. In immune cells, those alterations can result in diminished functional activities, such as reduction in neutrophils phagocytosis and killing capacity [2]. In addition, oxidative stress has been correlated with increased inflammation through the activation of pro-inflammatory signaling pathways [16] and with insulin resistance, which can further enhance the lipolytic state of transition dairy cows [19]. Overall, oxidative stress as an underlying factor for dysfunctional immune response and enhanced inflammation underscores the importance of this condition in the increased susceptibility of dairy cattle to health disorders, particularly during the transition period [2,11,20].

Antioxidant compounds can be synthesized by the body and are also naturally present in feedstuffs; however, for indoor animals fed low forage diets or especially during periods of high antioxidant demands such as the peripartal period, dietary supplementation is necessary to meet the increased requirements [21,22,23]. Vitamin E and selenium are the most widely used antioxidants included in dairy cattle diets, especially in the form of premixes added to the total mixed ration [11]. However, in the last few decades, there has been an increased interest in studying alternative natural compounds with antioxidant and anti-inflammatory properties as supplements for food-producing animals [24,25,26]. Besides being a source of antioxidants in the final products for the human diet, those natural compounds have the added benefit of being positively perceived by consumers [22,27].

Rosemary (*Salvia rosmarinus*), an herb from the Labiatae family, is known to have a particularly high concentration of phenolic diterpenes with antioxidant and antimicrobial properties [28]. Among those phenolic diterpenes, carnosic acid is the most abundant in rosemary leaves [29], and it has one of the highest antioxidant activity [30,31]. Carnosic acid presents a high reactivity toward ROS, and it acts as a scavenger that can eliminate ROS [28]. Moreover, carnosic acid has been shown to play a role in the activation of the PI3K/Akt/Nrf2 signaling pathway in human cells [32,33]. Nuclear factor erythroid-2 related factor 2 (Nrf2) is a transcription factor involved in the cellular response to oxidative stress, inducing the expression of several protective enzymes, e.g., glutathione peroxidase and superoxide dismutase [32]. Furthermore, carnosic acid has been shown to reduce the expression levels of pro-inflammatory cytokines in human and mice cells [34,35] and regulate fatty acid metabolism [36].

However, in ruminants, studies evaluating carnosic acid have been mainly correlated with an increase in meat quality through reduction of lipid oxidation [37,38,39]. Surprisingly, its potential use as a strategy to reduce the oxidative and metabolic stress of transition dairy cows remains *terra incognita*. Based on the above, we hypothesized that providing carnosic acid during early lactation might minimize the typical exposure to oxidative stress and attenuate the typical inflammatory response during peripartum while allowing cows to reach peak performance. The objective of the present study was to evaluate the effects of a compound found in rosemary plants (*Salvia rosmarinus*) named carnosic acid during the transition period of dairy cows.

## 2. Materials and Methods

### 2.1. Experimental Design and Treatments

The Institutional Animal Care and Use Committee (IACUC) of the South Dakota State University approved all the procedures for this study (protocol no. 2003-017A). The experiment was conducted from September to December 2020 at the South Dakota State University Dairy Research and Training Facility (Brookings, SD). Twenty late pregnant multiparous Holstein dairy cows were used in a randomized complete block design from −21 days prior to expected calving until 21 days in milk (DMI). Cows were blocked according to expected calving day, parity, and previous lactation milk yield, then assigned into one of two treatments. A total of 4 cows were removed from the experiment due to calving outside the range of −7 to 7 d relative to the expected calving date (*n* = 2), displaced abomasum (*n* = 1), and euthanasia due to low calcium/potassium at calving coupled with a lack of response to treatment (*n* = 1) (Table 1).

From day 1 to 3 after calving, cows received either a daily intravenous infusion of 500 mL of sterile saline solution (NaCl 0.9%; Saline; *n* = 8) or an infusion with carnosic acid at a rate of 0.3 mg/kg of BW (CA; *n* = 8). The complete dose of carnosic acid (Combi-Blocks, San Diego, USA, cat. number QC-4383) based on BW was supplied on a total volume of 500 mL of sterile saline solution. Treatments were infused into the external jugular vein before evening milking (17:00).

In rodents, pharmacokinetic studies have been performed using intravenous infusions with CA at a rate of 10 or 20 mg/kg [40,41]. To the authors’ knowledge, a carnosic acid pharmacokinetic study in ruminants has never been performed. Milk is the only body fluid that has been used to detect carnosic acid in ruminants supplemented with distilled rosemary leaves [42]. In this study Jordan, et al. [42], fed goats a basal diet supplemented with 0, 10, and 20% of the diet with a pellet containing barley and distilled rosemary leaves, and observed a carnosic acid concentration in milk ranging from 0.31 to 0.77 mg/kg. Therefore, given the limitations on pharmacokinetic data on carnosic acid in ruminants and the cost of procuring the CA couple with the large amount of CA needed to supply the corresponding dose for an adult dairy cow, a 0.3 mg/kg BW dose was adopted as the most feasible daily dosage from 1 to 3 d postpartum.

### 2.2. Animal Management

Cows were enrolled in the experiment from early September 2020 to early January 2021. Weather data from Mesonet at South Dakota State University (https://climate.sdstate.edu/) was used to evaluate the daily ambient temperature during the experimental period. Cows were fed using an individual gate system (American Calan, Northwood, NH, USA), and intakes were recorded daily. Diets were formulated using the CNCPS model contained within the Agricultural Modeling and Training Systems (AMTS) CattlePro diet-balancing software (version 4.16.1, AMTS LLC, Lansing, NY, USA) to meet the requirements of the average cow in the group (Table 2). Dry matter content of feed ingredients was determined once a week throughout the experiment, and diets were adjusted accordingly to maintain formulated DM ratios.

During the dry period, cows were housed in bedded pack pens. Immediately after calving, cows were reallocated in individual pens bedded with straw. On day 3, after calving, cows were moved to a lactation free-stall barn. Cows were fed once daily (6:00) and milked twice daily (6:30 and 18:00). Body weight was measured weekly for each cow in the morning (9:00). Body condition score (BCS) (scale 1 = thin to 5 = obese) was assigned by two individuals, and the average score was used for statistical analysis. All cows received the same close-up diet (1.46 Mcal of NEL/kg and 15.1% CP; Table 2) and lactation basal diet (1.75 Mcal of NEL/kg and 19.6% CP; Table 2), as a total mixed ration.

### 2.3. Blood Collection and Analyses

Blood was sampled from the coccygeal vein before morning feeding using a 20-gauge vacutainer needle (Becton Dickinson, Franklin Lakes, NJ) at –7, 2, 5, 7, 14, and 21 d relative to parturition. Blood was collected into evacuated tubes (BD Vacutainer, Becton Dickinson, Franklin Lakes, NJ) containing either serum clot activator or lithium heparin. After collection, tubes that contained lithium heparin were placed on ice, and tubes with serum clot activator were kept at 21 °C until centrifugation. Serum and plasma were obtained by centrifugation at 1300× *g* for 15 min at 21 °C and 4 °C, respectively. The aliquots were frozen at −80 °C until further analysis.

Blood samples were analyzed for biomarkers related to energy metabolism [i.e., glucose, β-hydroxybutyric acid (BHB), non-esterified fatty acids (NEFA)], muscle mass catabolism (i.e., urea and creatinine), inflammation (i.e., ceruloplasmin and haptoglobin), liver function [i.e., albumin, bilirubin, glutamic-oxaloacetic transaminase (GOT), γ-glutamyltransferase (GGT), cholesterol, and paraoxonase (PON)], and oxidative stress [i.e., myeloperoxidase (MPO), reactive oxygen metabolites (ROM), ferric reducing antioxidant power (FRAP), nitrates (NO_3_^−^), nitrites (NO_2_^−^), nitric oxide metabolites (NO_x_), and oxygen radical absorbance capacity (ORAC)]. Furthermore, we calculated the ratios between oxidants and antioxidant defenses, e.g., ROM/FRAP and ROM/ORAC. Those ratios provide an integrated oxidant status index, which seems to better assess changes in oxidative status during transition period [43,44].

Albumin, cholesterol, bilirubin, urea, creatinine, GOT, GGT, and glucose were analyzed using the IL Test purchased from Instrumentation Laboratory Spa (Werfen Co., Milan, Italy) in the ILAB 600 clinical auto-analyzer (Instrumentation Laboratory, Lexington, MA, USA), following the procedures described previously [45,46,47]. Haptoglobin was analyzed using the method described by Skinner, et al. [48], while ceruloplasmin was determined based on Sunderman and Nomoto [49], with modifications described by Jacometo, et al. [50]. Antioxidant potential was assessed as ferric reducing antioxidant power (FRAP) using a colorimetric method [51]. Paraoxonase, NO_x,_ NO_2_^−^_,_ and NO_3_^−^ were analyzed according to methods described by Trevisi, et al. [52]. Myeloperoxidase was determined via colorimetry based on the reaction of MPO contained in the plasma sample with hydrogen peroxide, which forms H_2_O and O^−^; the O^−^ dianisidine dihydrochloride, and electron donor, reacts with the O^−^, releasing H_2_O and a colored compound [50,53]. Non-esterified fatty acids and BHB were measured using kits from Wako (Chemicals GmbH, Neuss, Germany) and Randox (Randox Laboratories Ltd., Crumlin, UK), respectively, following the procedures described previously [45,53,54]. Finally, total antioxidants were assessed through the oxygen radical absorbance capacity (ORAC) assay. This method estimates the overtime antioxidant capacity to inhibit phycoerythrin hydroxyl radical damage [55].

### 2.4. Milk and Feed Samples

Total mixed ration samples were collected weekly and frozen at −20 °C after DM analysis until further nutrient profile analysis. Monthly composites were analyzed for contents of DM, CP, NDF, and ADF and NE_L_ was calculated using wet chemistry methods at a commercial laboratory (Dairy One; Ithaca, NY, USA).

Consecutive morning and evening milk samples were collected once weekly until 21 DMI. Composite milk samples were performed in proportion to milk yield at each milking, preserved (Broad Spectrum Microtabs II, Advanced Instruments, Norwood, MA, USA), and analyzed for fat, protein, lactose, solids, milk urea nitrogen (MUN), and somatic cell count (SCC) (Dairy One; Ithaca, NY, USA). Energy corrected milk (ECM) was calculated based on milk yield and milk sample analysis as follows: ECM = (12.82 × fat yield (kg)) + (7.13 × protein yield (kg)) + (0.323 × milk yield (kg)) [56].

Energy balance (EB) for each cow was calculated based on equations described previously [21]. The net energy intake (NE_I_) was determined based on daily DMI multiplied by NE_L_ density of the diet, and net energy of maintenance (NE_M_) was calculated as BW^0.75^ × 0.080. Requirements of net energy of lactation (NE_L_) were calculated as NE_L_ = (0.0929 × fat % + 0.0547 × protein % + 0.0395 × lactose %) × milk yield. The net energy requirement for pregnancy (NE_P_) was calculated as NE_P_ = ((0.00318 × day of gestation − 0.0352) × (calf birth weight/45))/0.218. The equation used to calculate prepartal EB (EB_PRE_; Mcal/d) was EB_PRE_ = NE_I_ − (NE_M_ + NE_P_) and EB_PRE_ (as % of requirements) = (NE_I_/(NE_M_ + NE_P_)) × 100. Finally, to calculate postpartal EB (EB_POST_), the equation used was EB_POST_ (Mcal/d) = NE_I_ − (NE_M_ + NE_L_) and EB_POST_ (as % of requirements) = (NE_I_/(NE_M_ + NE_L_)) × 100.

### 2.5. Statistical Analysis

The effects of carnosic acid blood biomarkers were evaluated separately at 2 d relative to parturition (during infusions) and from 5 to 21 d postpartum as residual effects. Performance data and residual effects were evaluated repeated measures using the MIXED procedure of SAS 9.4 (SAS Institute Cary NC, USA). The statistical model contained the effects of treatment, time (day or week), and their interactions as fixed effects, while the cow within treatment was considered as a random effect. Single time-point data were analyzed following the same model, without the time statement. Blood biomarkers were log-scale transformed if needed to comply with normal distribution of residuals.

Residual data on blood biomarkers from 5 to 21 d postpartum was unequally spaced; therefore, the SP(POW) covariance structure was used for this analysis. For the equally spaced measures, the covariance structure was chosen between first-order autoregressive and heterogeneous first-order autoregressive based on goodness of fit (smaller Akaike information criteria). Covariates, including previous 305 d milk yield, prepartum DMI, ambient temperature, and blood metabolites at –7 d relative to calving, were maintained in the model when *p* ≤ 0.20. Observations were considered outliers when Cook’s distance >0.50 and consequently excluded from the analysis. The CORR procedure of SAS was used to test the Pearson correlation coefficient (*r*) between milk performance and prepartum DMI, BW prepartum, and change in energy balance. The occurrence of health problems was analyzed using the FREQ procedure of SAS and interpreted based on Fisher’s exact test probabilities. However, none of the health issues observed in this experiment were affected (*p* ≥ 0.26) by treatment (Table 1). Statistical significance was declared at *p* ≤ 0.05 and tendencies at *p* ≤ 0.10.

## 3. Results

### 3.1. Peripartal DMI, BW, and BCS

Main effects and interactions for prepartum and postpartum BW, BCS, DMI, DMI as % of BW, and EB are presented in Table 3. Results show a Trt × T interaction (*p* ≤ 0.05) for prepartum DMI as % of BW and EB. However, no differences (*p* > 0.10) were observed between treatments at any given time point, and the interaction seems to be driven by changes in DMI as % of BW and EB from wk −2 to −1 observed for CA cows.

Moreover, CA cows had lower (*p* = 0.03) postpartum DMI as % of BW, while a trend (*p* = 0.06) for greater BW prepartum was observed compared to saline cows. In contrast, treatment effects (*p* > 0.10) were observed in postpartum BW. Body condition score and DMI were similar (*p* > 0.10) between treatment groups throughout the experimental period. Although EB was similar (*p* > 0.10) between treatments, further analysis showed that CA cows had a greater (*p* = 0.04) ($EB\;change=EB_{prepartum}-EB_{postpartum}$) than saline cows around parturition (−19.8 vs. −13.8 ± 1.9 Mcal/d).

### 3.2. Milk Production and Composition

**Figure 1:**
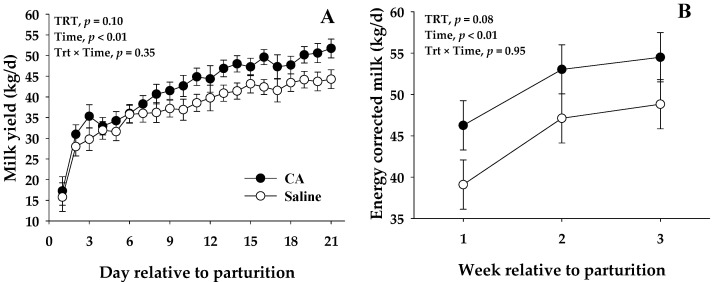
Milk yield (**A**) and energy corrected milk (**B**) for peripartal dairy cows infused from 1 to 3 d postpartum with 500 mL of sterile saline solution (Saline) or carnosic acid (CA) at a rate of 0.3 mg/kg BW supplied in a total volume of 500 mL of sterile saline solution. Values are means, and the standard errors are represented by vertical bars.

The main effects and interactions for postpartum production variables and milk composition are presented in Table 4. A trend (*p* ≤ 0.10) for greater milk yield (Figure 1), ECM, and milk efficiency as milk/DMI was observed in CA cows in comparison with saline cows. The CA cows produced 4.5 kg/d and 6.3 kg/d more milk and ECM than saline cows, respectively. Similarly to milk yield, a trend (*p* = 0.10) was observed for a greater milk protein yield in CA cows when compared to the saline group. Milk yield was negatively correlated with energy balance change around parturition (*r* = −0.63, *p* = 0.01) $\left(EB\;change=EB_{prepartum}-EB_{postpartum}\right)$. In contrast, milk yield and BW prepartum were not correlated in either CA cows (*r* = −0.24; *p* = 0.56) or saline (*r* = 0.27; *p* = 0.52) (Figure S1).

### 3.3. Blood Biomarkers

Immunometabolic effects of carnosic acid were evaluated at 2 d relative to calving and are present in Table 5. The CA cows had lower (*p* ≤ 0.05) concentrations of haptoglobin, paraoxonase, FRAP, and NO_2_^−^ than saline cows, while a trend (*p* = 0.07) for lower myeloperoxidase was observed in CA cows than saline cows.

**Figure 2:**
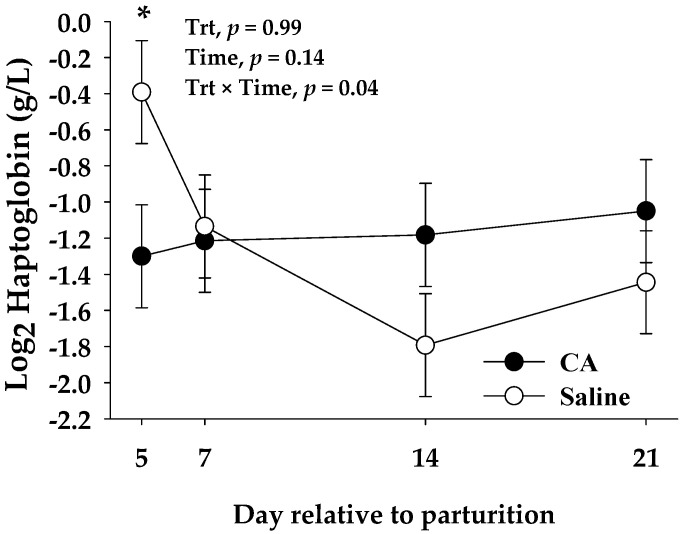
Residual effects of carnosic acid (CA) or saline intravenous infusions on blood biomarkers from 5 to 21 d relative to parturition for haptoglobin. Mean separations between treatments at a given time point were evaluated at a treatment × time interaction (*p* ≤ 0.10), and differences (*) were declared at *p* ≤ 0.05. Values are means and the standard errors are represented by vertical bars.

Residual effects of treatments on blood biomarkers are shown in Table 6. There was a Trt × T interaction (*p* ≤ 0.05) for haptoglobin, where CA cows had lower (*p* = 0.03) haptoglobin than saline cows at 5 d relative to parturition (Figure 2). A trend (*p* = 0.10) for lower NO_3_^−^ in CA cows than saline was observed from 5 to 21 d relative to parturition (Table 6).

## 4. Discussion

### 4.1. Performance Parameters

Antioxidants are frequently part of dairy cows’ diets and play a key role in minimizing harmful consequences of excessive production of reactive oxygen and nitrogen species, thereby improving their health status and reducing disease incidence [11,57]. Several studies have recently evaluated the effects of plant-based bioactive compounds with antioxidants properties as alternatives to conventionally available forms. In terms of animal productivity, performance responses when supplementing alternative plant-based antioxidants are not consistent among studies, most likely due to different properties of compounds, doses, animal species, among others [26,58]. For example, Oh, et al. [59] observed that oxidative stress markers were not affected when supplementing lactating dairy cows with *Capsicum* oleoresin. However, the authors observed increased ECM and neutrophil activity. In contrast, Mezzetti, et al. [60] reported positive effects in terms of liver function and redox balance when transition dairy cows were supplemented with *Aloe arborescens*; however, milk production was unaffected by treatment.

In the present study, we observed an effect of carnosic acid infusions to improve milk yield, ECM, and milk protein yield. Moreover, there was no difference in DMI between treatment groups, which resulted in greater milk efficiency in terms of milk yield/DMI. Although the effects of carnosic acid in dairy cows remain to be elucidated, dietary supplementation of rosemary plant (*Salvia rosmarinus*) to dairy goats and sheep has been evaluated in terms of performance but with inconsistent results. For instance, studies had reported an increase in milk yield when small ruminants were fed a diet supplemented with dried rosemary plant or extract [61,62,63], but this effect was not observed by others [42]. These benefits of dietary rosemary on lactation performance have been mainly associated with modifications in the ruminal environment by the interaction of compounds in rosemary (e.g., carnosic acid) with rumen microbiota. These studies suggest that the phenolic compounds found in rosemary leaves could inhibit protein degradation in the rumen, affect fatty acid metabolism during ruminal biohydrogenation, and enhance nutrient digestibility and ruminal fermentation [61,63].Therefore, a direct comparison between the effects of dietary rosemary plant or extract and intravenous infusion of carnosic acid is challenging. However, if further research confirms this effect, it will indicate that rosemary or rosemary compounds may influence milk yield in ruminants.

The lower DMI as % of BW aligned with greater EB change from pre- to postpartum observed in CA cows, suggesting a greater lipid mobilization. Even though the difference in EB postpartum did not reach statistical significance (Table 3). In the light of these results, we theorize that carnosic acid may influence liver lipid metabolism in transition dairy cows. In fact, carnosic acid has been shown to reduce hepatic lipid accumulation in mice models through down-regulation of *de novo* lipogenesis and up-regulation of fatty acid oxidation signals at the mRNA and protein level, including *PPARA* [36] and MAPK [64]. In transition dairy cows, increased fatty acid catabolism in the liver would induce the transmission of signals towards the brain satiety center, reducing feed intake, as explained by the hepatic oxidation theory [65]. Moreover, in this scenario, gluconeogenesis may be enhanced due to the higher availability of its precursors, e.g., pyruvate and oxalacetate. This theory could explain the lower DMI as % BW and the increase in milk production in CA cows. In fact, biomarkers for energy and liver function suggest that CA cows were able to cope with increased milk production without increased liver damage and risk of health disorders such as ketosis, as evidenced by BHB concentrations. However, we emphasize that the effects on hepatic metabolism here proposed are largely speculatory, and further studies are necessary to determine the specific mechanisms of how hepatocytes respond to carnosic acid under a peripartal condition in dairy cows.

### 4.2. Inflammation and Liver Function

Haptoglobin is a positive acute phase protein (APP) synthesized mainly by hepatocytes during an inflammatory process [66,67]. Higher haptoglobin concentrations are related to increased levels of pro-inflammatory cytokines, such as tumor necrosis factor alpha (TNF-α), IL-1, and IL-6 [53]. In dairy cows, haptoglobin levels are commonly increased during peripartum, with peak concentrations around the first week of lactation [53]. Therefore, this protein is a highly sensitive indicator of immune system activation and, as such, is a reliable biomarker of stress [4,66]. Additionally, haptoglobin is a major hemoglobin binding protein, limiting the oxidative tissue damage mediated by hemoglobin. The induction of haptoglobin typically associated with enhanced oxidative stress seems to be a mechanism to prevent excessive damage caused by free radicals [68].

In the present study, we observed a treatment effect on haptoglobin concentrations during the days of infusion (2 d) until 5 d relative to parturition (Table 5 and Figure 2, respectively). Several studies have observed the anti-inflammatory effects of carnosic acid [35,69,70]. This phenolic diterpene has been shown to significantly decreased the protein expression levels of various pro-inflammatory cytokines in serum and tissues, e.g., TNF-α, IL-1β, and IL-6 [34], through the suppression of the jun-N-terminal kinase (JNK), nuclear factor κB (NF-κB) and signal transducer and activator of transcription 3 (STAT3) pathways [35,71]. Concentrations of haptoglobin in the days following calving observed in our study suggest a lower inflammatory status in CA cows, which agrees with previous studies using different animal models [26].

Haptoglobin and PON are APP produced mainly by the liver. However, while haptoglobin is a positive APP, PON is characterized as a negative APP, meaning its hepatic synthesis is impaired during inflammation [4,53]. In the plasma, PON is transported in association with high-density lipoproteins, and it exerts an important antioxidant function by hydrolyzing lipid hydroperoxides generated during oxidative stress [72,73]. Moreover, the decreased PON activity following parturition observed, especially in high producing dairy cows, is associated with intense lipomobilization and fat deposition in the hepatocytes, related to liver damage or dysfunction [73].

In the present study, lower concentrations of PON were observed for CA cows than saline cows at 2 d relative to parturition (58 vs 67 U/mL). However, there was no difference between treatments from 5 to 21 d (83.1 vs 92.1 ± 5.3 U/mL). The reduction of PON activity at 2 d could be an indication of increased inflammation, intense lipomobilization, reduced levels of blood HDL, oxidative stress, or a combination of those [53]. However, literature suggests that, regardless of treatment, PON activity observed throughout our study was higher than those associated with impaired liver function or adverse health conditions after parturition [53,66]. For example, Bionaz, et al. [53] evaluated the relationship between PON activity during the first month of lactation with health problems, inflammatory conditions, and liver function. Cows classified as lower quartile (43.8 ± 12.7 U/mL) based on PON activity presented lower milk yield, higher blood ROM, and greater occurrence of serious infections when compared with cows in the upper quartile (92.0 ± 19.8 U/mL). Moreover, our results regarding the other biomarkers for liver function support the idea that carnosic acid infusion from 1 to 3 d relative to parturition did not negatively affect the liver of the experimental animals.

### 4.3. Oxidative Stress

Around the 2 d relative to parturition, we also observed a treatment effect on plasma MPO, in which CA cows had lower concentrations than saline cows. MPO is a lysosomal peroxidase enzyme mainly stored in the azurophilic granules of neutrophils and to a lesser degree in primary lysosomes of monocytes [74,75]. Upon immune cell activation, the enzyme is released extracellularly and into phagosomal compartments. In the presence of H_2_O_2_ and a halide (chloride, bromide, or thiocyanate), MPO catalyzes the formation of reactive oxygen intermediates, including hypochlorous (HOCl) [74,76]. MPO is an important component of the host innate immune system against invading microorganisms. Thus it has been used as an inflammation biomarker [75].

Reactive oxygen species produced by MPO have been implicated as mediators of the host oxidative tissue damage and cellular dysfunction [74,75]. Results found in our study are in agreement with previous authors, which observed an effect of carnosic acid to decrease the activity of MPO in mice immune cells during acute lung injury [77] and acute colitis [78]. According to those studies, carnosic acid may attenuate oxidative stress by suppressing the production of pro-inflammatory cytokines, immune cells activation, and migration. Lower levels of MPO aligned with haptoglobin results, suggesting lower inflammatory condition in CA cows than saline cows around the infusion days.

In contrast with our initial hypothesis, blood ROM was not affected by treatment. Those results are in contrast with previous in vitro studies that observed a lower accumulation of ROS when different cell lines were treated with carnosic acid [79,80,81]. de Oliveira, et al. [33] found lower ROS concentrations when neuroblastoma cells were treated with carnosic acid during induced neurotoxicity. However, the authors observed no differences when cells were not challenged. Overall, literature suggests that the effect of carnosic acid on the production of reactive oxygen compounds might be dose-dependent, and cellular conditions may have an important role in its effectiveness. Alternatively, the lack of effect on ROM due to CA infusion could be attributed to CA attenuating ROS production at a phagocytic cell level, but not sufficient to affect ROS being produced during the high metabolic state at the onset of lactation. Moreover, the limitation of in vivo experiments, especially in ruminants, makes it particularly challenging to correlate our findings with a specific biological mechanism.

The high complexity of the oxidant defense system and dynamics between prooxidants and antioxidants among different tissues makes it challenging to quantify and infer the whole-organism overall redox balance accurately. The individual quantification of specific antioxidants does not provide a complete picture of the antioxidant capacity in a given sample since multiple mechanisms act synergically to counterbalance oxidative stress [11,51]. In this context, different analytic methods, i.e., FRAP and ORAC, were developed to estimate antioxidant activity of a certain sample. An increase in FRAP plasma values indicates a greater need for neutralizing ROM production, while higher ORAC denotes greater protection produced by antioxidants in plasma [46].

The CA cows had a lower FRAP at 2 d relative to parturition when compared to saline cows. However, no differences were observed for ORAC, ROM/FRAP, or ROM/ORAC. Different studies with rats have shown that plasma concentrations of carnosic acid decline rapidly after intravenous infusions, while gastrointestinal absorption results in longer plasma retention [40,41]. In the present study, blood samples were collected around 12 h after intravenous infusions, which might help explain why results of plasma antioxidant activity and oxidative status were mostly unaffected by carnosic treatment. Furthermore, studies with carnosic acid in ruminant species are scarce, and the effects of its supplementation on plasma FRAP or ORAC are currently unknown. Jordan, et al. [38] evaluated the level of transfer of two typified rosemary extracts (carnosic acid and carnosol) in lamb tissues after dietary supplementation. Carnosic acid, especially at 1:1 ratio with carnosol, increased muscle and liver FRAP, although plasma FRAP was not evaluated in that study.

Carnosic acid treatment reduced levels of NO_2_^−^ around the period of infusions (2 d postpartum). Moreover, a residual effect of treatment was observed in terms of NO_3_^−^ from 5 to 21 d relative to parturition. Nitrite (NO_2_^−^) and nitrate (NO_3_^−^) are stable products of nitric oxide (NO), and the measurement of these oxidation products is used to estimate NO production in biological fluids [46,82]. Nitric oxide is an important free radical synthesized by immune and endothelial cells; it is involved in many biological functions, including intracellular communication, vasodilatation, and inflammation [83,84,85]. At physiological levels, its toxicity is generally limited [84]. However, at high concentrations, NO is rapidly oxidized to reactive nitrogen oxide species (RNOS), e.g., nitrogen dioxide (NO_2_) and dinitrogen trioxide (N_2_O_3_), which in turn induce cell toxicity through modification of enzymes, signaling proteins, and transcription factors [86]. Different studies have observed decreased levels of NO in the presence of carnosic acid, which was associated with lower levels of the inducible NO synthase enzyme (iNOS) [70,87]. Overall, our results indicate that carnosic acid may have decreased NO synthesis, suggesting lower inflammation and a lower risk of oxidative damage.

## 5. Conclusions

The findings of this study revealed that carnosic acid promoted positive responses on inflammation and oxidative stress biomarkers of around the period of infusions. In addition, carnosic acid contributed to an increase in lactation performance, although the mechanisms responsible for this response remain unclear. Pharmacokinetics studies are necessary to further understand how carnosic acid is metabolized in ruminants and how fast this occurs in order to estimate optimal dosage and carnosic acid supplementation through its plant-based source, rosemary. Overall, the results presented here describe the potential benefits of providing antioxidants such as carnosic acid during the peripartal period of dairy cows.

## Figures and Tables

**Table 1:** Frequency of occurrence of health problems in peripartum dairy cows receiving an intravenous infusion of carnosic acid (CA) or saline (Saline) from −21 relative to parturition through 21 DIM.

	Treatment	
Event	CA	Saline
Total animals	10	10
Subclinical ketosis ^1^	5	4
Clinical ketosis ^2^	0	2
Retained placenta ^3^	0	1
Displaced abomasum	0	1
Metritis ^4^	1	0
Pneumonia	1	0
Total excluded cows ^5^	2	2

^1^ Defined as cows having 1.4 to 2.9 mmol/L ketone concentration in blood, detected using a Precision Xtra analyzer (Abbott Labs). Treatment administrated was oral propylene glycol and injection of vitamin B complex; ^2^ Defined as cows having > 2.9 mmol/L ketone concentration in blood, detected using a Precision Xtra analyzer (Abbott Labs). Treatment administrated was oral propylene glycol and injection of vitamin B complex; ^3^ Defined as fetal membranes retained > 24 h postpartum; ^4^ Foul-smelling, watery-consistency uterine discharge after calving; ^5^ Cows excluded from the experiment due to calving outside the range of −7 to 7 d relative to the expected calving date (CA, *n* = 1; Saline, *n* = 1), displaced abomasum (Saline, DIM = 18, *n* = 1), euthanasia due to low calcium/potassium at calving coupled with a lack of response to treatment (CA, DIM = 0, *n* = 1).

**Table 2:** Ingredient composition of diets during the close-up (−21 d to expected calving) and early lactation periods (21 d relative to calving).

	Diet	
Component	Close-up	Lactation
**Ingredient, % of DM**		
Corn silage	36.9	32.5
Alfalfa hay	-	7.6
Alfalfa haylage	-	12.1
Grass hay	24.6	-
Wheat straw	12.4	-
Soybean meal	11.5	5.7
Cottonseed	-	6.7
Molasses	-	5.0
Dry cow grain mix ^1^	14.6	-
Lactating cow grain mix ^2^	-	30.4
**Chemical analysis**		
DM, %	46.7	47.6
NEL, Mcal/ kg DM	1.46	1.75
CP, % DM	15.1	19.6
NDF, % DM	43.7	31.8
ADF, % DM	27.4	17.7
Starch, % DM	15.9	28.2
DCAD, mEq/100 g	−12.3	-

^1^ Dry cow grain mix contained (as % DM): distillers grain dry (24.8), soy hulls (21.4), biochlor (18.5), limestone Ca (14.0), magnesium sulfate 7H_2_O (3.5), calcium phosphate 21% (3.5), reashure choline (2.8), calcium chloride dihy (2.3), magnesium oxide 54% (2.3), calcium sulfate dihyd (1.5), chromium propionate 0.04% (1.0), DV nutritek (0.9), JPW dairy vitamin premix 8298.V05 (0.9), salt white (0.7), vitamin E 20000 IU/lb (0.7), JPW dairy TM premix 8298.E04 (0.7), DTX binder (0.4), rumensin 90 g/lb (0.1); ^2^ Lactating cow grain mix contained (as % DM): corn grain ground fine (66.1), soy best (14.8), distillers grain dry (7.4), limestone Ca (2.7), sodium bicarbonate (2.8), energy booster 100 (2.2), salt white (1.16), urea (0.90), magnesium oxide 54% (0.55), calcium phosphate 21% (0.33), JPW dairy vitamin premix 8298.V05 (0.29), JPW dairy TM premix 8298.E04 (0.29), DV nutritek (0.22), vitamin E 20000 IU/lb (0.11), DTX binder (0.11), rumensin 90 g/lb (0.02), biotin 1% (0.02).

**Table 3:** Differences between treatments groups during prepartum and main residual effects of intravenous infusion of carnosic acid (CA) or saline solution (Saline) on BW, BCS, DMI, DMI as % of BW, and energy balance.

	Treatment			*p*-Value		
Parameter	CA	Saline	SEM ^1^	Trt	Time	Trt × T ^2^
**Prepartum**						
Body weight, kg	873.3	801.6	25.0	0.06	0.01	0.34
Body condition score	3.70	3.75	0.08	0.65	0.74	0.24
Dry matter intake, kg/d	15.3	13.2	0.92	0.12	<0.01	0.41
Dry matter intake, % BW	1.73	1.69	0.13	0.82	<0.01	0.04
Energy balance, Mcal/d	6.10	4.49	1.56	0.47	<0.01	0.05
**Postpartum**						
Body weight, kg	735.5	739.9	7.64	0.71	<0.01	0.19
Body condition score	3.55	3.54	0.04	0.88	<0.01	0.13
BW change ^3^, %	−13.0	−11.6	0.92	0.33	<0.01	0.22
BCS change ^3^, %	−4.8	−5.2	1.06	0.77	<0.01	0.26
Dry matter intake, kg/d	18.6	20.2	0.72	0.15	<0.01	0.70
Dry matter intake, % BW	2.48	2.85	0.11	0.03	<0.01	0.33
Energy balance, Mcal/d	−13.51	−9.53	1.82	0.14	0.02	0.19

^1^ Largest standard error of the mean is shown; ^2^ Interaction of treatment and time (day or week) relative to calving; ^3^ Percent change from the average of 3 weeks prepartum.

**Table 4:** Main residual effects of intravenous infusion of carnosic acid (CA) or saline solution (Saline) on milk production and composition parameters during 21 d in milk.

	Treatment			*p*-Value		
Parameter	CA	Saline	SEM ^1^	Trt	Time	Trt × T ^2^
Milk yield, kg/d	41.8	37.3	1.76	0.10	<0.01	0.35
Energy corrected milk ^3^, kg/d	51.3	45.0	2.35	0.08	<0.01	0.95
Milk efficiency, Milk/DMI ^4^	2.37	2.04	0.12	0.06	0.02	0.56
Milk efficiency, ECM/DMI	2.75	2.47	0.17	0.25	0.03	0.11
**Milk Composition**						
Fat, %	5.07	5.26	0.23	0.56	<0.01	0.78
Fat yield, kg/d	2.11	1.89	0.12	0.19	0.13	0.82
Protein, %	3.52	3.53	0.14	0.96	<0.01	0.60
Protein yield, kg/d	1.45	1.27	0.07	0.10	0.18	0.93
Lactose, %	4.69	4.74	0.05	0.49	<0.01	0.56
Solids, %	14.35	14.62	0.25	0.47	<0.01	0.70
Milk urea nitrogen, mg/dL	12.71	12.19	0.92	0.70	0.73	0.88
Log-transformed SCC ^5^	4.93	5.11	0.14	0.40	0.08	0.79

^1^ Largest standard error of the mean is shown; ^2^ Interaction of treatment and time (day or week) relative to calving; ^3^ Energy corrected milk (ECM), calculated as (12.82 × fat yield (kg)) + (7.13 × protein yield (kg)) + (0.323 × milk yield (kg)); ^4^ DMI: dry matter intake; ^5^ SCC: somatic cell count.

**Table 5:** Effects of intravenous infusion of carnosic acid (CA) or saline solution (Saline) on blood biomarkers related to energy metabolism, inflammation, liver function, muscle body mass, metabolism, and oxidative stress in dairy cows at 2 d relative to parturition.

	Treatment			*p*-Value
Parameter	CA	Saline	SEM ^1^	Trt
**Energy metabolites**				
Glucose, mmol/L	4.10	4.03	0.11	0.68
BHB, mmol/L	0.83	0.82	0.12	0.98
NEFA, mmol/L	0.83	0.65	0.13	0.35
**Inflammation**				
Ceruloplasmin, µmol/L	2.30	2.41	0.07	0.38
Haptoglobin ^2^, g/L	0.67	1.06	0.16	<0.01
**Liver function**				
Albumin, g/L	33.46	33.97	0.49	0.48
Cholesterol, mmol/L	1.74	1.84	0.09	0.42
Paraoxonase, U/mL	58.03	67.38	2.61	0.03
Bilirubin ^2^, µmol/L	5.17	5.21	0.42	0.99
AST, U/L	119.9	105.3	8.20	0.23
GGT ^2^, U/L	17.39	16.34	0.09	0.48
**Muscle mass catabolism**				
Urea, mmol/L	5.39	5.28	0.27	0.80
Creatinine, µmol/L	94.25	88.27	2.97	0.21
**Oxidative stress**				
FRAP ^2^, µmol/L	140.1	163.1	0.06	0.02
Myeloperoxidase, U/L	485.3	519.6	12.8	0.07
ROM, mg H_2_O_2_/100mL	15.88	16.73	0.52	0.28
NO_2_^–^, µmol/L	2.63	4.58	0.35	<0.01
NO_3_^–^, µmol/L	21.85	19.62	1.13	0.19
NO_x_, µmol/L	23.9	23.65	0.87	0.84
ORAC, µg/mL	12.50	13.06	0.34	0.25
ROM/FRAP	0.11	0.10	0.01	0.44
ROM/ORAC	1.25	1.32	0.06	0.40

^1^ Largest standard error of the mean is shown; ^2^ Data were log-transformed before statistics. The standard errors of the means associated with log-transformed data are in log scale.

**Table 6:** Residuals effects of intravenous infusion of carnosic acid (CA) or saline solution (Saline) on blood biomarkers related to energy metabolism, inflammation, liver function, muscle body mass, metabolism, and oxidative stress in dairy cows from 5 to 21 d relative to calving.

	Treatment			*p*-Value		
Parameter	CA	Saline	SEM ^1^	Trt	Time	Trt × T ^3^
**Energy metabolites**						
Glucose, mmol/L	3.87	4.02	0.08	0.20	0.03	0.88
BHB, mmol/L	1.06	0.99	0.21	0.83	0.73	0.90
NEFA, mmol/L	0.63	0.63	0.09	0.99	<0.01	0.88
**Inflammation**						
Ceruloplasmin, µmol/L	2.88	2.99	0.12	0.55	0.28	0.90
Haptoglobin ^2^, g/L	0.44	0.44	0.17	0.99	0.14	0.04
**Liver function**						
Albumin, g/L	35.51	34.71	0.33	0.11	0.03	0.53
Cholesterol, mmol/L	2.92	3.11	0.13	0.31	<0.01	0.37
Paraoxonase, U/mL	83.08	92.07	5.33	0.25	0.10	0.79
Bilirubin ^2^, µmol/L	3.46	3.66	0.20	0.78	<0.01	0.79
AST, U/L	149.2	122.9	13.43	0.19	0.21	0.34
GGT ^2^, U/L	23.92	20.25	0.16	0.32	0.06	0.40
**Muscle mass catabolism**						
Urea, mmol/L	5.01	5.06	0.23	0.87	0.37	0.51
Creatinine, µmol/L	84.83	82.35	1.42	0.25	<0.01	0.71
**Oxidative stress**						
FRAP ^2^ ,µmol/L	128.0	132.5	0.09	0.71	<0.01	0.12
Myeloperoxidase, U/L	447.4	436.1	19.9	0.70	0.01	0.95
ROM, mg H_2_O_2_/100mL	17.61	18.40	0.69	0.44	0.10	0.35
NO_2_^–^, µmol/L	5.78	5.90	0.42	0.85	<0.01	0.92
NO_3_^–^, µmol/L	21.71	23.07	0.58	0.10	0.01	0.91
NO_x_, µmol/L	28.25	28.21	0.72	0.97	<0.01	0.96
ORAC, µg/mL	12.56	12.23	0.58	0.70	0.95	0.97
ROM/FRAP	0.14	0.15	0.01	0.86	<0.01	0.69
ROM/ORAC	1.48	1.56	0.09	0.53	0.74	0.56

^1^ Largest standard error of the mean is shown; ^2^ Data were log-transformed before statistics. The standard errors of the means associated with log-transformed data are in log scale; ^3^ Interaction of treatment and days relative to calving.

## Data Availability

Data are contained within the article or supplementary files.

## Supplementary Materials

The following are available online at https://www.mdpi.com/article/10.3390/antiox10091478/s1, Figure S1. Correlation between body weight prepartum and milk yield for peripartal dairy cows infused with 500 mL of sterile saline or carnosic acid at a rate of 0.3 mg/kg BW supplied in a total volume of 500 mL of sterile saline solution.
