# Supplementary material for: An Exploration of the Effects of an Early Postpartum Intravenous Infusion with Carnosic Acid on Physiological Responses of Transition Dairy Cows
Source: Antioxidants (Basel). 2021 Sep 16;10(9):1478. doi: 10.3390/antiox10091478 (PMC8466393; doi:10.3390/antiox10091478)
Supplement: Supplementary file 1 [file antioxidants-10-01478-s001.zip › antioxidants-1344644-supplementary.pdf]

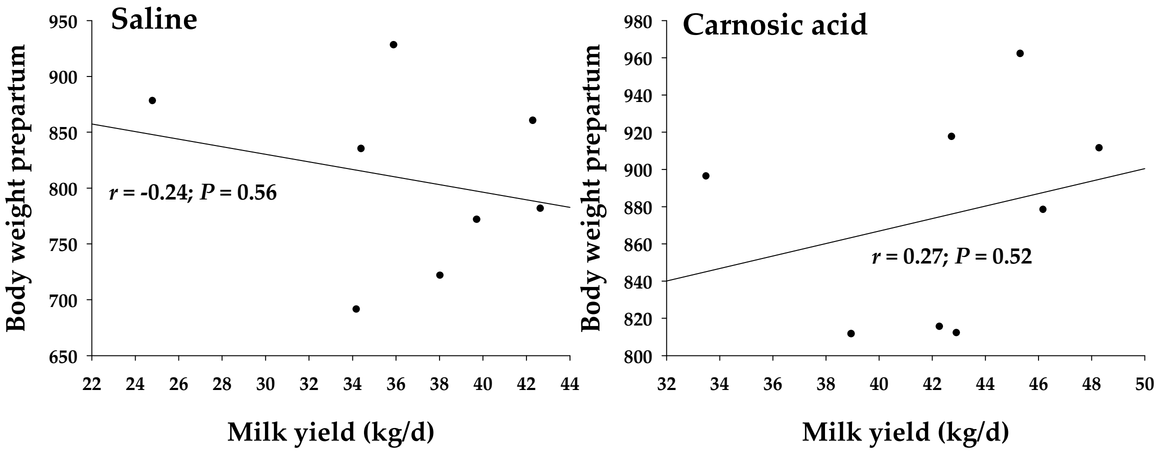

Figure S1. Correlation between body weight prepartum and milk yield for peripartal dairy cows infused with 500 mL of sterile saline or carnosic acid at a rate of 0.3 mg/kg BW supplied in a total volume of 500 mL of sterile saline solution.
